# Determination of Electrolytes in Critical Illness Patients at Different pH Ranges: Whom Shall We Believe, the Blood Gas Analysis or the Laboratory Autoanalyzer?

**DOI:** 10.1155/2019/9838706

**Published:** 2019-07-15

**Authors:** Christopher Hohmann, Roman Pfister, Kathrin Kuhr, Julia Merkle, Julian Hinzmann, Guido Michels

**Affiliations:** ^1^Department III of Internal Medicine, Heart Center, University Hospital of Cologne, Cologne, Germany; ^2^Institute of Medical Statistics and Computational Biology, Faculty of Medicine and University Hospital Cologne, University of Cologne, Cologne, Germany; ^3^Department of Cardiothoracic Surgery, Heart Center, University Hospital of Cologne, Cologne, Germany

## Abstract

**Introduction:**

The determination of the electrolytes sodium and potassium is essential in critical care. In daily clinical practice, both the blood gas analyzer (ABG) and the laboratory autoanalyzer (AA) are generally applied. However, there is still uncertainty regarding the convergence of the prementioned assays, and data about the comparability dependent on the pH value are still lacking.

**Materials and Methods:**

One hundred samples from intensive care unit patients with a range in pH values between 7.20 and 7.49 were evaluated in this retrospective cohort study. All patients suffered an infarct-related cardiogenic shock and were intubated and not under therapeutical hypothermia at the time of blood collection. We used scatter plots to compare different distributions of sodium and potassium values between the methods. Comparability of the analyses was assessed using the Bland–Altmann approach, and intraclass correlations (ICC) as estimates of interrater reliability were calculated.

**Results:**

The mean potassium level measured on ABG was 4.33 mmol/L (SD 0.48 mmol/L), and the value obtained using the AA was 4.40 mmol/L (SD 0.55 mmol/L). A Bland–Altman comparison for total potassium measurements revealed that the limits of agreement were small (−0.241 to 0.391 mmol/L). Total ICC displayed a very good correlation of 0.949. For sodium, we found average values of 140 mmol/L (SD 5.20 mmol/L) in the AA and 140 mmol/L (SD 5.80 mmol/L) in the ABG assessment. Contrarily, the Bland–Altman comparison for sodium displayed that the 95% limits of agreement were very wide (−5.99 to 6.59 mmol/L) for total measurements as well as in every pH subgroup. Total ICC only reached a value of 0.830.

**Conclusion:**

Data from our single-center study indicate that urgent and vital decisions based on potassium measurements can be made by trusting the value obtained on the ABG machine irrespective of pH values.

## 1. Introduction

Due to their fundamental effects on cellular functioning and metabolic processes, the accurate quantification of the electrolytes sodium and potassium is essential in clinical practice and especially in intensive care unit patients [1, 2].

There are generally two approaches applied in daily use. On the one hand, electrolytes are routinely measured from serum by central laboratory autoanalyzers (AAs); however, the time between the blood drawing and the achievement of results depends on various factors such as processing, transport time, and laboratory sample analysis [3]. Obviously, such delay may compromise the treatment of critically ill patients. On the other hand, arterial blood gas (ABG) analyzers have a wide application in nearly all emergency departments and intensive care units (ICUs) due to the opportunity to obtain data quickly and to respond with prompt treatment [4]. However, current data regarding the comparability and validity between the two processes are ambiguous. For instance, some recent studies revealed considerable differences between the ABG and AA measurements [5, 6]. Furthermore, ABG machines are not primarily designed to provide accurate and precise results for electrolytes, but rather blood gas analyses. Because of this fact, ABG analyses are sometimes not trusted for clinical decision making with respect to electrolyte measurement. More importantly, especially in the event of significant shifts in pH values, which physiologically lead to an electrolyte displacement and penetrability of membranes, valid data are generally lacking. As a result, previous studies have shown that although the majority of clinicians consider point-of-care (POC) testing to be a useful method to obtain rapid results, less than a half would rely on such results and prefer to wait for central laboratory confirmation before making important clinical decisions [7].

In our retrospective study, we investigated for the first time whether sodium and potassium ion concentrations measured with the central laboratory autoanalyzer or arterial blood gas analyzer in critical illness patients differ depending on the pH value.

## 2. Materials and Methods

A retrospective observational study of a consecutive cohort of adult patients between 44 and 56 years of age admitted to the intensive care unit of the Cardiology Department at the University hospital of Cologne, Germany, was conducted for a period of 3 months between 01 July and 01 October 2017. All patients suffered an infarct-related cardiogenic shock and were intubated and not under therapeutical hypothermia at the time of blood collection.

We included only patients whose paired blood samples were routinely collected from an arterial catheter as it is established as an internal standard in our cardiac intensive care unit. Complementary measurements of electrolytes in the central laboratory are generally part of our daily blood diagnostics.

100 samples were analyzed with a range in pH values of 19 samples being 7.20–7.29, 41 samples with a pH value between 7.30 and 7.39, and 40 samples with pH levels between 7.40 and 7.49.

Blood samples were taken subsequently from the arterial catheter after disposing the first two milliliters in order to avoid dilution effects. The serum sample was obtained by withdrawing 3.0 ml of blood in a plain vacutainer. For the arterial blood gas analyzer, 2.0 ml of blood was collected in commercially available plastic arterial blood gas syringes (PICO 50 Radiometer, Radiometer Medical ApS, Bronshoj, Denmark; 2.0 ml volume, no recommended draw volume, coated with 80 IU electrolyte-balanced heparin).

ABG samples received in heparinized syringes were processed immediately for electrolytes in the ICU, and the samples collected in vacutainers were transported and analyzed in the central clinical laboratory within a maximum of 1 hour after collection.

Electrolytes were measured with a benchtop AA (Modular ISE 900-Modul, Roche Diagnostics, Mannheim, Germany) and with an ABG analyzer (ABL800 FLEX, Radiometer GmbH, Krefeld, Germany). For electrolytes, both instruments work on the principle of ion-selective electrodes (ISEs). However, the measurement is performed on diluted plasma (indirect) for the former and on whole-blood samples (direct) for the latter. Generally, the results are comparable to those afforded by the recognized reference method flame photometry [8]. The ABG was calibrated automatically and routinely according to the manufacturer's recommendation.

The reference ranges for sodium and potassium were taken as 135–145 mmol/L and 3.5–5.2 mmol/L, respectively. According to the United States Clinical Laboratory Improvement Amendments (US CLIA), a maximum difference of 4.0 mmol/L in sodium levels and 0.5 mmol/L in potassium measurements is generally tolerated and acceptable [9]. All samples were collected by intensive care personnel.

## 3. Statistical Analysis

We used scatter plots to compare different distributions of sodium and potassium values between the methods. Comparability of the analyses was assessed using the Bland–Altmann approach, and intraclass correlations (ICC) as estimates of interrater reliability were calculated [10]. Limit of agreement was defined as the mean bias ± standard deviation (SD). Statistical analysis was performed using SPSS version 24.0 (SPSS Inc., Chicago, USA).

## 4. Results

**Figure 1:**
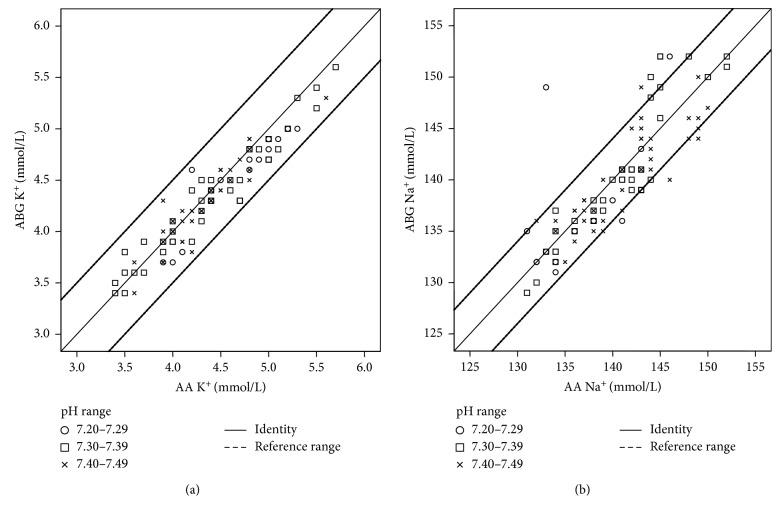
Scatter plots presenting total measurements with ABG and AA for potassium (a) and sodium (b). Deviations in measurements of potassium with AA and ABG were all within the reference range of a maximum difference of 0.5 mmol/L overall and separately for distinct pH value ranges (a). For sodium, analysis demonstrated several variations above and below a threshold value of 4.0 mmol/L for all pH ranges (b). Solid line: identity between the measuring methods. Dashed lines: maximum tolerated difference according to the US CLIA.

**Figure 2:**
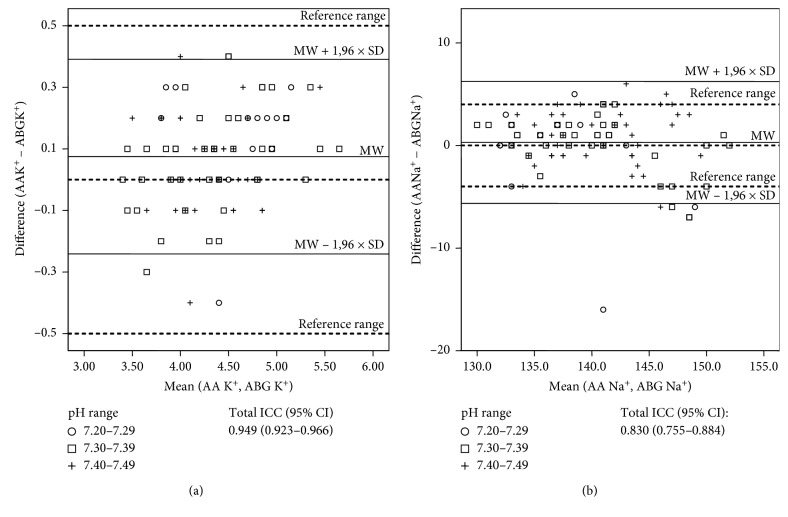
Bland–Altman comparison of AA and ABG measurements for potassium and sodium. The plot of difference of 2 methods against the mean of 2 methods for potassium and sodium measurements, respectively. Bland–Altman plots are provided for total measurements showing the 95% limits of agreement. Comparison of AA and ABG for total potassium measurement results revealed that the limits of agreement were −0.241 to 0.391 mmol/L (a). These limits were clinically acceptable and the total ICC displayed a very good correlation of 0.949. In contrast, Bland–Altman comparison of the ABG and AA data for sodium revealed that the 95% limits of agreement were very wide (−5.99 to 6.59 mmol/L) for total measurements as well as in every pH subgroup, which was not clinically acceptable (b). Total ICC only reached a value of 0.830. Solid line: mean difference as well as maximum tolerated difference according to the US CLIA. Dashed lines: mean bias plus or minus standard deviation.

Differences of sodium and potassium in the ABG and AA at different pH values are summarized in Table 1. The mean potassium level measured on ABG was 4.33 mmol/L (SD 0.48 mmol/L), and the value obtained using the AA was 4.40 mmol/L (SD 0.55 mmol/L). Deviations in measurements of potassium with AA and ABG were all within the reference range of a maximum difference of 0.5 mmol/L overall and separately for distinct pH value ranges (Figure 1(a)). A Bland–Altman comparison of AA and ABG for total potassium measurement results revealed that the limits of agreement were −0.241 to 0.391 mmol/L. As shown in Figure 2(a), these limits were clinically acceptable and the total ICC displayed a very good correlation of 0.949. For subgroup analyses, the samples with a blood pH of 7.20–7.29 showed a mean potassium level of 4.65 mmol/L in AA (SD 0.47 mmol/L) and 4.52 mmol/L in ABG measurements (SD 0.46 mmol/L). 41 samples with pH values between 7.30 and 7.39 displayed average potassium measurements of 4.40 mmol/L (SD 0.62 mmol/L) and 4.32 mmol/L (0.55 mmol/L) in the AA and ABG, respectively. Moreover, 40 samples with a blood pH of 7.40–7.49 revealed mean values of 4.31 mmol/L (SD 0.39 mmol/L) in the AA and 4.26 mmol/L (SD 0.39 mmol/L) in the ABG group. In the subgroup assessment for different pH values in the Bland–Altman comparison, limits of agreement for potassium were correspondingly small in every pH range and ICCs again showed a good conformity with the highest correspondence for pH values between 7.30 and 7.39.

For sodium, analysis demonstrated several variations above and below a threshold value of 4.0 mmol/L for all pH ranges (Figure 1(b)). In its entirety, we found average values of 140 mmol/L (SD 5.20 mmol/L) in the AA and 140.06 mmol/L (SD 5.80 mmol/L) in the ABG assessment. When analyzing the 3 subgroups depending on different pH values, the mean sodium levels in samples with pH ranges of 7.20–7.29 were 137 mmol/L (SD 6.47 mmol/L) in the ABG and 136 mmol/L (SD 4.62 mmol/L) in the AA measurement, respectively. For pH values between 7.30 and 7.39, the mean sodium level in the ABG was 140 mmol/L (SD 6.37 mmol/L) and in the AA analyses was 141 mmol/L (SD 5.15 mmol/L). Measurements of sodium levels in samples with pH values of 7.40–7.49 revealed a mean value of 141 mmol/L (SD 4.93 mmol/L) in the AA and 141 mmol/L (SD 4.62 mmol/L) in the ABG analyzer. Additionally, the Bland–Altman comparison of the ABG and AA data revealed that the 95% limits of agreement for sodium were very wide (−5.99 to 6.59 mmol/L) for total measurements as well as in every pH subgroup, which was not clinically acceptable. Total ICC only reached a value of 0.830 (Figure 2(b)). Interestingly, the subgroup of pH values between 7.30 and 7.39 again corresponded most exactly within the different methods. There was only little correspondence of measurements in case of a pH value between 7.20 and 7.29.

## 5. Discussion

Critical illness patients at the intensive care unit require more frequent monitoring of laboratory values and especially electrolytes. Point-of-care (POC) analyzers are regularly used by physicians and medical staff in the area of critical care and emergency medicine due to several advantages such as rapid processing times and lower costs [10]. Moreover, this analytical method is not affected by serum protein levels, which are known to be low especially in critically ill patients [11]. Despite the many benefits, there is frequently uncertainty regarding the comparability and validity with central laboratory tests.

In this study, we aimed to compare measurements of the electrolytes sodium and potassium depending on different blood pH values using a benchtop AA as well as an ABG analyzer. Previous studies reported significant differences in Na^+^ levels when using these two different types of measurements and hence point out potential risks in therapeutic decisions [12, 13]. Additionally, due to marked differences also in chloride levels, Moramitsu et al. indicated different assessments not only in electrolyte levels but consequently further in the calculation of the anion gap [5]. Conversely, in relation to their findings, Jain et al. suggested the possibility of a reasonable and reliable decision based on serum potassium levels yielded by using an ABG analyzer [14].

These previously observed differences between the electrolyte levels measured using an AA and ABG analyzer may be explained on the basis of a combination of factors such as sample transport, dilution determinants, or instrument calibration. For instance, it is well known that ISE-based analysis devices from different manufacturers yield sodium/potassium levels that differ by 2–5% [15]. Moreover, for various types of heparin in blood gas syringes, a preanalytical bias in electrolyte concentrations was demonstrated. For this reason, likewise in our study, preheparinized dry and balanced syringes are recommended for ABG sampling [16]. Another influential factor might be sample hemolysis due to prolonged transportation times and laboratory diagnostics. As the potassium concentration in erythrocytes is 25 times higher than in normal plasma, sample hemolysis can exert a strong influence on result reliability, and clinically meaningful variations of potassium and sodium were already observed in specimens displaying mild or almost undetectable hemolysis by visual inspection [17]. However, there are no definite indications on the degree of lysis responsible for relevant interference. Furthermore, hemolyzed samples are a rather frequent occurrence in laboratory practice, with a prevalence of up to 3.3% in all routine samples presented to a clinical laboratory [18, 19]. In our sample series, at least no measurable hemolysis could be detected.

Consistent with previous results in literature, our data indicate that potassium levels obtained from ABG machines are comparable with benchtop AA analyzers and hence are overall reliable for making sufficient clinical decisions. With regard to sodium, we found individual deviations up to 16 mmol/L between the ABG and AA samples. Hence, the limits of agreement were very wide and ICC indicated only a limited interrater reliability. The US CLIA demands a maximum difference of 4.0 mmol/L in sodium levels and 0.5 mmol/L in potassium measurements, compared to target values [8]. For potassium, this requirement was fully met in the current data whereas sodium did not reach this standard in our test series.

In addition to the known differences between AA and ABG, this is the first study providing further information about the comparability of these measurement techniques even in case of divergences in blood pH values since acute disturbances in acid-base equilibrium are known to result in changes in plasma electrolyte concentrations. Regarding potassium, limits of agreement were all within the reference range defined by the US CLIA, independently of the pH values, and hence indicate a good conformity between the different methods. For sodium, the limits of agreement within the subgroups were all very wide and outside the accepted range of 4 mmol/L.

As a result, in case of emergency situations due to electrolyte imbalances, it appears to be acceptable to rely on ABG analyzer measurements for potassium and to adjust therapy from the values so measured. In contrast, particularly in case of relevant hyponatremia or hypernatremia, where possible, corresponding reference values from the central laboratory should be awaited.

These results are generally in line with previously published data [15]. However, amongst other studies, Budak et al. found out that even potassium test results obtained using an ABG and an AA differ and the data thus cannot be used interchangeably in clinical practice [20].

Considering these previous ambiguous findings in literature and the data in our study, determining the concordance of electrolyte levels obtained by using ABG analyzers and those measured in the central laboratory is of pronounced importance. As instrument types, materials, and calibration procedures may differ among hospitals, in-house studies should be conducted for the assessment of the reliability of point-of-care analyses and probably need to be repeated on a regular basis to provide reliable calibration. If necessary, a correction factor needs to be determined in case of relevant deviations in order to minimize measurement errors.

## 6. Limitations of the Study

This study was a single-center study with a relatively small number of samples, especially in the patient group with pH values between 7.20 and 7.29. All blood samples were taken to the central laboratory within one hour after collection. However, we cannot totally exclude technical problems causing inappropriate collection, prolonged transportation times, rough handling, incorrect temperature, and delayed laboratory diagnostics. Sample hemolysis can exert a strong influence on result reliability especially for potassium values. However, as mentioned above, in particular, the results for potassium fully met the criteria of the US CLIA. Hence, we are reasonably confident that hemolysis did not affect our data in an important manner.

## 7. Conclusions

Measurements of potassium in the ABG and AA yielded a good conformity between the different methods independent of pH values, whereas the determination of sodium displayed significant deviations in all subgroups. Therefore, it appears to be acceptable that urgent and vital decisions based on potassium measurements can be made by trusting the value obtained with the ABG machine. However, as instrument types and calibration methods differ between different clinics, in-hospital studies regarding the agreement between the assays are essential and probably need to be repeated on a regular basis to provide reliable calibration. In doubt, a simultaneously follow-up sample must be sent to the central laboratory for confirmation.

## Figures and Tables

**Table 1:** Differences in measurements of sodium and potassium in the ABG and AA at different pH values.

	Sodium (Na^+^) (AA-ABG) (mmol/l)		Δ Na^+^ (AA-ABG) (mmol/l)	Potassium (K^+^) (AA-ABG) (mmol/l)		Δ K^+^ (AA-ABG) (mmol/l)
pH 7.20–7.29 (*n* = 19)	136 ± 4.62	137 ± 6.47	3.07 ± 4.18	4.65 ± 0.47	4.52 ± 0.46	0.19 ± 0.11
pH 7.30–7.39 (*n* = 41)	140 ± 5.15	140 ± 6.37	1.90 ± 1.89	4.40 ± 0.62	4.32 ± 0.55	0.14 ± 0.11
pH 7.40–7.49 (*n* = 40)	141 ± 4.93	140 ± 4.62	2.29 ± 1.56	4.31 ± 0.39	4.26 ± 0.39	0.12 ± 0.11
pH 7.20–7.49 (*n* = 100)	140 ± 5.20	140 ± 5.80	2.24 ± 2.27	4.40 ± 0.55	4.33 ± 0.48	0.14 ± 0.11

## Data Availability

The data used to support the findings of this study are available from the corresponding author upon request.
